# International code of conduct for genomic and health-related data sharing

**DOI:** 10.1186/1877-6566-8-1

**Published:** 2014-06-14

**Authors:** Regulatory and Ethics Working Group, Global Alliance for Genomics & Health, Sumio Sugano

**Affiliations:** Department of Medical Genome Sciences, Graduate Scool of Frontier Sciences, The university of Tokyo, 4-6-1, Shirokanedai, 108-8639 Minato-ku, Tokyo, Japan

**Keywords:** Data Sharing, Universal Declaration, Research Data System, Foundational Principle, International Ethical Guideline

## Preamble

The sharing of scientific, genomic and health-related data for the purpose of research is of key importance in ensuring continued progress in our understanding of human health and wellbeing. New challenges raised by international, collaborative research require a principled and practical framework that brings together researchers, regulators, funders, patient groups, information technologists, and consortia to share data. Such a framework will facilitate global science and responsible research conduct.

This International Code of Conduct provides guidance for the responsible sharing of genomic and health-related data. In particular, it is guided by Article 27 of the 1948 *Universal Declaration of Human Rights*. Article 27 guarantees the rights of every individual in the world “*to share in scientific advancement and its benefits*”, to freely engage in responsible scientific inquiry, and at the same time “*to the protection of the moral and material interests resulting from any scientific…production of which [a person] is the author*.” Article 27 has been expanded by other enforceable international conventions and national laws, regulations, codes and policies set out in Appendix 1.

This Code establishes a set of foundational principles and guidelines for responsible research conduct and oversight of research data systems, guided by the enforceable human rights of privacy, non-discrimination, and procedural fairness. The Code also: (a) interprets the right to enjoy the benefits of scientific progress and its applications as being the right to engage in responsible scientific inquiry, access and share genomic and health-related data across the translation continuum, from basic research through practical applications; and (b) applies the right to benefit from the protection of the moral and material interests resulting from scientific productions to health-related research by developing rights for data producers.

The value of this Code is that it: offers political and legal dimensions that reach beyond the moral appeals of bioethics and provides a more robust governance framework for genomic and health-related data sharing; speaks to groups and institutions, not just individuals; stresses the progressive realization of duties, urging action by governments, industry, funders, and researchers to create an environment for responsibly sharing data; and fosters responsible research in health by offering stronger protection in critical areas.

## Purpose and interpretation

The purpose of this Code is to provide a principled and practical framework for the sharing of genomic and health-related data. This Code should be interpreted in good faith. This Code is to be understood as a whole and the Foundational Principles and Guidelines are to be understood as complementary and interrelated. Each Foundational Principle and Guideline is to be considered in the context of the other Foundational Principles and Guidelines, as appropriate and relevant in the circumstances.

The primary goals of this Code are to:

1.1. Protect and promote the welfare, rights, and interests of groups and individuals who provide their data;
1.2. Provide guidance and benchmarks for accountability;
1.3. Complement laws and regulations on privacy and data protection, as well as codes for the ethical governance of research;
1.4. Foster responsible data sharing and oversight of research data systems;
1.5. Establish a framework for greater international data sharing cooperation, collaboration, and good governance;
1.6. Serve as a dynamic instrument that can grow and change in response to future developments in the science and practices of genomic and health-related data sharing; and
1.7. Be a tool for the evaluation of research by research ethics committees.

## Application

This Code may be adopted and implemented by organizations and bodies involved in genomic and health related data sharing.

This Code applies to:

2.1. Researchers, research participant and patient communities;
2.2. Research organizations;
2.3. Universities, colleges and research institutes;
2.4. National and regional research ethics and other government bodies;
2.5. Research ethics committees and data access committees;
2.6. Clinics, hospitals, health care centers and institutes;
2.7. Research funding agencies;
2.8. Professional research organizations;
2.9. Industry;
2.10. Information technologists and research data system providers; and
2.11. Other bodies or individuals requesting, accessing, managing or otherwise using genomic and health-related data.

## Foundational principles

The Foundational Principles of this Code guide responsible data sharing. They also facilitate compliance with the obligations set by international and national law and policies.

### Foundational principles for responsible data sharing

- Promote Health and Wellbeing.
- Respect Individuals, Families and Communities.
- Advance Research and the Fair Distribution of Benefits.
- Foster Trust, Integrity and Reciprocity.

## Guidelines and core elements

These Guidelines include core elements that aim to practically apply the Foundational Principles to individuals and organizations involved in the sharing of genomic and health-related data. The Guidelines should be interpreted in a proportionate manner that acknowledges different levels of risk and community cultural practices. This Code applies to data that has been consented to for use and/or approved therefor by competent authorities. Adherence to the Guidelines does not preclude further consideration and development of additional elements.

### Guidelines and core elements for responsible data sharing

1. Transparency
  1.1. Developing clearly defined and accessible information on the purposes, processes, procedures and governance frameworks for data sharing.
  1.2. Providing information on the purpose, collection, use and exchange of genomic and health-related data, including: data transfer to third parties, international transfer of data, terms of access, re-identifiability and limits to anonymity or confidentiality of data, return of results, commercial involvement, and proprietary claims.
  1.3. Implementing procedures for fairly adjudicating requests for data access and exchange.

2. Accountability
  2.1. Putting in place systems for data sharing that are compliant with this Code and for tracking the chain of data exchange.
  2.2. Identifying processes to identify and manage conflicts of interest.
  2.3. Implementing mechanisms for handling complaints and enforcing this Code, and identifying and managing breaches of the Code.

3. Data Security and Quality
  3.1. Storing and processing the data collected, used and transferred that is accurate, verifiable, unbiased, and current, so as to enhance their interoperability and replicability while preserving their long-term searchability and integrity.
  3.2. Ensuring feedback mechanisms on the utility, quality, security, value, consistency and annotations on data, with a view to improving their quality and interoperability and re-use by others.
  3.3. Installing strict data security measures to prevent unauthorized access, data loss and misuse, and to ensure that all users in the chain of data exchange are in compliance with these measures.

4. Privacy, Data Protection and Confidentiality
  4.1. Complying with applicable privacy and data protection regulations, along with assurances that confidentiality and privacy are appropriately protected when data are collected, used and exchanged. Depending on the nature of the data, different levels of privacy and data protection may be necessary.
  4.2. Informing individuals, families and communities about the use and exchange of data relating to them, depending on the nature of the data.

5. Minimizing Harm and Maximizing Benefits
  5.1. Conducting data sharing with a view towards minimizing harms and maximizing benefits to not just those who contribute their data, but also to society and health care systems as a whole, particularly where data pertains to people based in more disadvantaged parts of the world.
  5.2. Considering the realistic harms and benefits of data sharing on individuals, families and communities, including opportunity costs.
  5.3. Undertaking a proportionate assessment of the benefits and risks of harm in data sharing, which is periodically monitored according to the reasonable foreseeability of such harms and benefits.

6. Recognition and Attribution
  6.1. Designing systems of data sharing with a view towards recognition and attribution that are meaningful and appropriate to the medium or discipline concerned and which provide due credit and acknowledgement of all who contributed to the results.
  6.2. Extending recognition and attribution equally to secondary or downstream uses and applications. All parties should act in good faith to ensure that the connections to original sources of data are maintained in the public record to the extent permissible by law.

7. Sustainability
  7.1. Addressing projects and systems for sharing genomic and health-related data at the earliest stage so as to ensure the sustainability of the data generated for future use, including their archiving and identifiability.

8. Accessibility and Dissemination
  8.1. Ensuring accessibility of data for approved research and data sharing purposes.
  8.2. Promoting collaborative partnerships and data sharing that can generate maximum value, along with the harmonization of deposit, management and access procedures and use as a means to promote accessibility.
  8.3. Publishing and disseminating research results, both positive and negative, depending on the nature of the data. Dissemination of research results should be conducted in a way that promotes broad access and minimizes obstacles to data sharing.
  8.4. Making data and research results widely available in a manner that promotes scientific collaboration and reproducibility.

## Implementation mechanisms and amendments

5.1. Persons and entities listed in section 2 of this Code should take all appropriate measures, whether of a legislative, administrative or other character, to give effect to the Foundational Principles and Guidelines set out in this Code in accordance with the international law of human rights and should, by means of all appropriate measures, promote their implementation.
5.2. Any persons and entity listed in section 2 of this Code may propose one or more amendments to the present Code by communicating the amendments to the Regulatory and Ethics Working Group of the Global Alliance for Genomics and Health (the ‘REWG’). The Regulatory and Ethics Working Group shall publicly circulate such amendments for comments and eventual adoption.
5.3. The REWG, in collaboration with biomedical, patient advocacy, and ethical and policy organizations and committees, will track the application of this Code, and routinely review its provisions in order to keep the Code continuously up-to-date with new advances in basic research and technology, as well as ethical and legal developments.

## Appendix 1

### Foundational human rights instruments

*Universal Declaration of Human Rights (UN 1948) (Article 27)

*International Covenant on Economic, Social and Cultural Rights (UN 1966) (Article 15)

### Ethical and legal codes and policies guiding data sharing behavior

- Constitution of the World Health Organization (WHO 1946)
- Bermuda Principles on Human Genome Sequencing (1996)
- Universal Declaration on the Human Genome and Human Rights (UNESCO 1997)
- The Convention on Human Rights and Biomedicine (Council of Europe 1997)
- Statement on DNA Sampling: Control and Access (HUGO 1998)
- Statement on Human Genomic Databases (HUGO Ethics Committee 2002)
- Declaration of Ethical Considerations regarding Health Databases (WMA 2002)
- International Ethical Guidelines for Biomedical Research Involving Human Subjects (CIOMS, WHO 2002)
- Budapest Open Access Initiative (2002)
- Sharing Data from Large-scale Biological Research Projects: A System of Tripartite Responsibility (Fort Lauderdale Statement, 2003)
- International Declaration on Human Genetic Data (UNESCO, IBC 2003)
- European Society of Human Genetics: Data Storage and DNA Banking for Biomedical Research (ESHG 2003)
- Universal Declaration on Bioethics and Human Rights (UNESCO 2005)
- Additional Protocol to the Convention on Human Rights and Biomedicine, concerning Biomedical Research (Council of Europe 2005)
- Recommendation Rec (2006) 4 of the Committee of Ministers to Member States on Research on Biological Materials of Human Origin (Council of Europe 2006)
- OECD Principles and Guidelines for Access to Research Data from Public Funding (OECD 2007)
- International Ethical Guidelines for Epidemiological Studies (CIOMS, WHO 2008)
- 2008 Best Practices for Repositories: Collection, Storage, Retrieval and Distribution of Biological Material for Research (ISBER 2008)
- Recommendations from the 2008 International Summit on Proteomics Data Release and Sharing Policy (Amsterdam Principles, 2008)
- Guidelines for Human Biobanks and Genetic Research Databases (OECD 2008, 2009)
- Toronto Statement on Prepublication Data Sharing (2009)
- Joint Statement by Funders of Health Research (2011)
- Responsible Conduct in the Global Research Enterprise: A Policy Report (InterAcademy Council 2012)
- Declaration of Helsinki (WMA 2013)
- Guidelines governing the Protection of Privacy and Transborder Flows of Personal Data (OECD 2013)

